# Supporting Equity and Inclusion of Deaf and Hard-of-Hearing Individuals in Professional Organizations

**DOI:** 10.3389/feduc.2021.755457

**Published:** 2021-10-15

**Authors:** Julia Jones Huyck, Kelsey L. Anbuhl, Brad N. Buran, Henry J. Adler, Samuel R. Atcherson, Ozan Cakmak, Robert T. Dwyer, Morgan Eddolls, Fadhel El May, Juergen-Theodor Fraenzer, Rebekah Funkhouser, Mathilde Gagliardini, Frederick J. Gallun, Raymond L. Goldsworthy, Samir Gouin, Joseph Heng, Ariel Edward Hight, Zina Jawadi, Damir Kovacic, Rachit Kumar, Santosh Kumar, Stacey R. Lim, Chengeng Mo, Lisa S. Nolan, Alexandra Parbery-Clark, Dominic V. Pisano, Valluri R. Rao, Robert M. Raphael, Lina A. J. Reiss, Nathaniel J. Spencer, Stephen J. Tang, Viral D. Tejani, Emma D. Tran, Mikaeel Valli, Greg D. Watkins, Rachel V. Wayne, Lindsey R. Wheeler, Stephanie L. White, Victor Wong, M. Caroline Yuk, J. Tilak Ratnanather, Peter S. Steyger

**Affiliations:** 1 Speech Pathology and Audiology, Kent State University, Kent, OH, United States; 2 Center for Neural Science, New York University, New York, NY, United States; 3 Oregon Hearing Research Center, Oregon Health & Science University, Portland, OR, United States; 4 Center for Hearing and Deafness, State University of New York at Buffalo, Buffalo, NY, United States; 5 University of Arkansas for Medical Sciences, Little Rock, AR, United States; 6 New Jersey Institute of Technology, Newark, NJ, United States,; 7 Vanderbilt University Medical Center, Nashville, TN, United States; 8 University Medical Center Göttingen, Göttingen, Germany,; 9 Bioglobe GmbH, Hamburg, Germany; 10 Department of Audiology, Nova Southeastern University, Fort Lauderdale, FL, United States; 11 Institut de l’Audition, Institut Pasteur, INSERM, Paris, France; 12 Keck School of Medicine, University of Southern California, Los Angeles, CA, United States; 13 McGill University, Montréal, QC, Canada; 14 Department of Medicine, University of Chicago, Chicago, IL, United States; 15 NYU Langone Medical Center, New York University, New York, NY, United States; 16 David Geffen School of Medicine, University of California, Los Angeles, Los Angeles, CA, United States; 17 Faculty of Science, University of Split, Split, Croatia; 18 Perelman School of Medicine at the University of Pennsylvania, Philadelphia, PA, United States,; 19 National Centre for Cell Science, S. P. Pune University Campus, Pune, India; 20 Department of Audiology, Central Michigan University, Mount Pleasant, MI, United States; 21 Faculty of Education, The University of Hong Kong, Hong Kong, Hong Kong SAR, China; 22 Wolfson Centre for Age-Related Diseases, King’s College London, London, United Kingdom; 23 Swedish Neuroscience Institute, Seattle, WA, United States; 24 Department of Anesthesiology, Tufts Medical Center, Boston, MA, United States; 25 Independent researcher, Saratoga, CA, United States; 26 Department of Bioengineering, Rice University, Houston, TX, United States; 27 Air Force Research Laboratory, Wright-Patterson Air Force Base, Dayton, OH, United States,; 28 Legacy Good Samaritan Medical Center, Portland, OR, United States; 29 Department of Otolaryngology-Head and Neck Surgery, University Hospitals Cleveland Medical Center, Cleveland, OH, United States; 30 Department of Otolaryngology-Head and Neck Surgery, Case Western Reserve University, Cleveland, OH, United States; 31 Department of Otolaryngology - Head and Neck Surgery, University of Minnesota, Minneapolis, MN, United States; 32 Institute of Medical Science, University of Toronto, Toronto, ON, Canada; 33 School of Biomedical Engineering, The University of Sydney, Darlington, NSW, Australia; 34 University Health Network, Toronto, ON, Canada; 35 School of Communication Sciences and Disorders, University of Memphis, Memphis, TN, United States; 36 Weill Cornell Medical College, New York, NY, United States; 37 Department of Chemistry and Biochemistry, University of Alabama, Tuscaloosa, AL, United States; 38 Department of Biomedical Engineering, Johns Hopkins University, Baltimore, MD, United States; 39 Translational Hearing Center, Department of Biomedical Sciences, School of Medicine, Creighton University, Omaha, NE, United States

**Keywords:** Diversity & Inclusion, disability, hearing loss, professional organisations, peer mentoring

## Abstract

Disability is an important and often overlooked component of diversity. Individuals with disabilities bring a rare perspective to science, technology, engineering, mathematics, and medicine (STEMM) because of their unique experiences approaching complex issues related to health and disability, navigating the healthcare system, creatively solving problems unfamiliar to many individuals without disabilities, managing time and resources that are limited by physical or mental constraints, and advocating for themselves and others in the disabled community. Yet, individuals with disabilities are underrepresented in STEMM. Professional organizations can address this underrepresentation by recruiting individuals with disabilities for leadership opportunities, easing financial burdens, providing equal access, fostering peer-mentor groups, and establishing a culture of equity and inclusion spanning all facets of diversity. We are a group of deaf and hard-of-hearing (D/HH) engineers, scientists, and clinicians, most of whom are active in clinical practice and/or auditory research. We have worked within our professional societies to improve access and inclusion for D/HH individuals and others with disabilities. We describe how different models of disability inform our understanding of disability as a form of diversity. We address heterogeneity within disabled communities, including intersectionality between disability and other forms of diversity. We highlight how the Association for Research in Otolaryngology has supported our efforts to reduce ableism and promote access and inclusion for D/HH individuals. We also discuss future directions and challenges. The tools and approaches discussed here can be applied by other professional organizations to include individuals with all forms of diversity in STEMM.

## INTRODUCTION

A diverse scientific workforce leads to increased creativity and productivity by introducing a wider range of perspectives, experiences, and skill sets (Lindsay et al., 2018; Tilghman et al., 2021). Diversity is conventionally considered in terms of race, ethnicity, country of origin, linguistic background, socioeconomic background, gender, and/or sexual orientation. Despite its high prevalence in society, disability is often overlooked as a form of diversity (Santuzzi and Waltz, 2016; Dennissen et al., 2018; Gould et al., 2021). While the importance of cultural and ethnic diversity is widely acknowledged, it is important to consider the contribution that individuals with disabilities can make in science, technology, engineering, mathematics, and medicine (STEMM) (Bellman et al., 2018). As with other diverse groups, people with disabilities are underrepresented in STEMM (National Science Foundation, National Center for Science and Engineering Statistics, 2021).

Individuals with disabilities offer a unique perspective drawn from their experiences of living with a disability that can guide novel and innovative scientific progress or improve medical care for people with that particular disability. They frequently have extensive experience navigating healthcare settings as patients, utilizing creativity to overcome accessibility challenges, managing limited time and resources, and advocating for themselves and others with disabilities. This experience translates to valuable attributes such as better communication skills, persistence, empathy, planning, creativity, and adaptability (Hewlett, 2017; Accenture, 2018; Kristjansdottir et al., 2018; Lindsay et al., 2018; DeFelice, 2019; Kulkarni, 2020). Individuals with disabilities often know how to use assistive technology to increase their productivity (Hatton, 2014; Kulkarni, 2020), and some types of disability or neurodiversity have common attributes that might be helpful in and of themselves. For example, employees who are Deaf or hard of hearing (D/HH) may be less distracted by background noise and conversations (Hatton, 2014). Businesses who actively recruit and support individuals with disabilities often see higher revenue and better employee retention than businesses that do not (Accenture, 2018; Lindsay et al., 2018).

**FIGURE 1 |:**
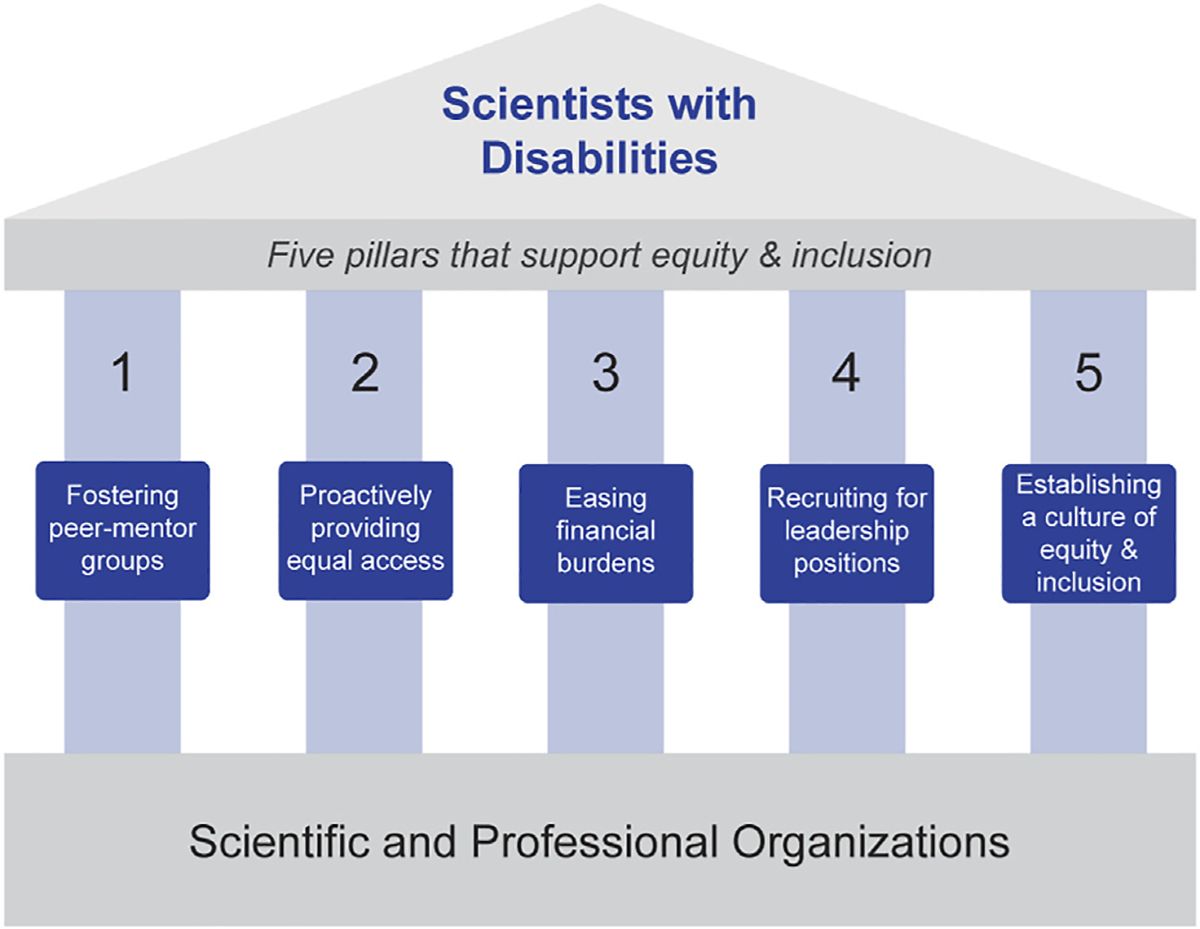
The five pillars that Professional and Scientific Organizations can use to support equity and inclusion for scientists with disabilities. The Association for Research in Otolaryngology (ARO) has implemented these building blocks which have been successful in supporting their deaf and hard-of-hearing (D/HH) members.

As defined by the United Nations’ Convention on the Rights of Persons with Disabilities (2007), “Persons with disabilities include those who have long-term physical, mental, intellectual or sensory impairments in which interaction with various barriers may hinder their full and effective participation in society on an equal basis with others.” Scientists with disabilities face several hurdles to equity and inclusion (Brown, 2016; Brown et al., 2018; Brown and Leigh, 2018; Inckle, 2018; De Picker, 2020). In the context of STEMM education, disability equity refers to justice in the way that people with disabilities are treated, such that they have the same likelihood for educational and career success as individuals who are not disabled. Disability equity also extends to interpersonal relationships, where individuals with disabilities should be treated with the same respect as individuals who are not disabled. Inclusion of individuals with disabilities means giving them physical, social, and financial access to the same opportunities as individuals without disabilities, often through the use of accommodations. Physical access refers to whether a person with a disability can enter, move around in, and function in a physical space and whether they are able to interact with physical materials (e.g., posters, lab equipment). For D/HH individuals, physical access can be facilitated by using microphones, providing services like captioning, cued language transliteration, or sign language interpretation, and by providing quiet environments for conversations. Social access refers to being able to attend and participate in the informal social interactions (e.g., conversations, dinners, social hours) that can lead to research collaborations, employment, and leadership opportunities. Social access is often a challenge for D/HH individuals who may be unable to hear in noisy conference halls or restaurants, or who may be exhausted from trying to hear at a conference and thus unable to attend evening events. Finally, financial considerations can limit both physical and social access. Many individuals with disabilities expend significant financial resources on health care and assistive technology. For these reasons, they sometimes cannot afford conference registration fees, travel costs, or educational expenses. Many interactions that are critical to career success occur at professional organizations’ academic or scientific conferences. Given the key role professional organizations play in nurturing scientists and facilitating career growth for trainees, we believe these organizations can foster diversity by supporting efforts for recruiting and supporting individuals of diverse backgrounds, including those with disabilities. Indeed, there has been recent interest in how scientific conferences can be more inclusive (Oswald and Ostojic, 2020; Tzovara et al., 2021). We propose five pillars (see Figure 1) that organizations can use to better support their diverse members: 1) fostering peer-mentor groups, 2) proactively providing equal access, 3) easing financial burdens, 4) recruiting for leadership positions, and 5) establishing a culture of inclusion and equity. By adopting these pillars, professional organizations can lead by example, familiarizing their members with equitable treatment and inclusion of individuals with disabilities and those from other diverse backgrounds.

We are a group of more than 110 deaf and hard-of-hearing (D/HH) engineers, scientists, and clinicians, most of whom are active in clinical practice and/or auditory research (Adler et al., 2017). Our network was initially formed in 1992 at a professional society meeting for the Association for Research in Otolaryngology (ARO) and has continued to grow based on shared experiences at those meetings and in other STEMM environments. Typically, our group makes up 1% or less of attendees at professional conference meetings. Diversity networks such as ours have the potential to promote equity and inclusion if they directly address systemic inequalities in organizations, in addition to advancing career development and building community (Dennissen et al., 2018). We have worked with the organizational leadership of various professional societies to implement successful, practical strategies for improving accommodations, raising awareness, and promoting academic, research, and career development opportunities for diverse trainees. We discuss how one organization, ARO, has fostered a more inclusive and equitable environment for researchers with disabilities and provide five guidelines for other scientific or professional organizations to consider.

## MODELS OF DISABILITY

Our peer mentorship network for D/HH individuals endorses a biopsychosocial view of hearing loss (defined below) that incorporates medical and social models of disability. While these disability models are not mutually exclusive, each model has distinct approaches to disability.

*Medical models* of disability address the physical differences associated with specific diagnoses and focus on preventing, curing, remediating, or accommodating the physical underpinnings of a given disability (Pelka, 2012). For D/HH individuals, this might involve genetic counseling about hereditary hearing loss, treatments to preserve or restore inner ear function, auditory rehabilitation through the use of hearing devices (e.g., hearing aids, cochlear implants), and promoting the individual use of assistive technology such as remote microphone technology and smartphone apps to provide automatic speech recognition and transcription. As scientists, engineers, and clinicians who study hearing and live with hearing loss, much of our academic labor falls into this category.

*Social models* of disability assert that some of the issues experienced by individuals with disabilities arise from the presence of societal or environmental barriers (Samaha, 2007). For deafness and hearing loss, addressing these barriers might include installing amplification systems that are compatible with hearing aids and cochlear implants, providing real-time captioning for all lectures and meetings by default, and providing interpreting and transliteration services (e.g., sign language interpreters, cued language transliterators, oral transliterators). Proponents of social models of disability often feel the language used to write and talk about disability should use “person-first language” (e.g., researchers with disabilities). This approach is meant to emphasize the personhood of individuals with disabilities and to avoid defining individuals and groups based only on their disability (Dunn and Andrews, 2015).

One type of social model, the diversity model, acknowledges that society and the environment can be inaccessible and exclusionary and also argue that people with disabilities form an important cultural group that contribute to society through their identity as disabled people (Andrews, 2020). This type of model recognizes that disabled people are culturally valuable because of their experiences of living with disability, and not because they are able to “overcome”—or succeed in spite of—their disability. For example, extensive advocacy by the D/HH community during the 1960s through 1980s led to ubiquitous captioning of movies and television broadcast shows. Today, the majority of viewers who now use captioning for their own benefit do not have a hearing loss (Ofcom, 2006).

The diversity model perspective has long been held by the Deaf community (commonly designated by the capital “D” to indicate that it is specific cultural group differentiated from the broader D/HH community) who view hearing loss as a key part of their personal identity and often use sign language as a primary form of communication (Padden and Humphries, 1988). More broadly, disability culture includes individuals with any and all disabilities and encompasses values, arts, and political stances in addition to experiences of shared discrimination and prejudice (see Andrews, 2020; Mackelprang and Salsgiver, 2016; or Brown, 2002, for reviews). For example, the American Deaf community has developed poetry and storytelling techniques that leverage linguistic features unique to American Sign Language (Bahan, 2006; Sutton-Spence and de Quadros, 2014).

Diversity models of disability contributed to the rise of the field of critical disability studies, which focuses on how different diverse identities can intersect with disabilities and draws parallels between the disability rights movements and other civil rights movements (Andrews et al., 2019; Ginsberg and Rapp, 2017). This field has led to our current understanding of ableism, defined as the conscious or unconscious prejudice and discrimination towards disabled people (Andrews, 2020). Ableism takes many forms and can be harmful regardless of whether it stems from ignorance, negative attitudes such as viewing disabled people as burdensome or vulnerable, or from attitudes which on the surface seem more positive, such as viewing disabled people as inspirational or heroic (Andrews, 2020). For example, media profiles of successful disabled individuals in academia seldom acknowledge that the individual’s disability can be an asset and not just something to “overcome” (e.g., Terry, 2019). Often, these narratives either ignore the challenges associated with disability entirely or assert that a scientist with a disability is noteworthy because they succeeded in spite of their disability. Ideally, these profiles would mention the challenges of being disabled and also include information on how their disability helped them to be more successful or a better scientist. Further, inspirational videos and articles of deaf children’s reactions when their cochlear implant is first activated may objectify them and fail to provide adequate background regarding realistic benefits of the device and remaining challenges post-implantation.

Proponents of diversity models and the field of disability studies often advocate for the use “identity-first language” (e.g., D/HH scientists) which enables disabled individuals to express belonging and pride as members of a disabled community and to define what it means to be disabled instead of letting it be defined by negative stereotypes (Dunn and Andrews, 2015). The American Psychological Association (2020) states that both person-first and identity-first language are appropriate, and that authors should consider the preferences of the people who are being written about. In this article, we have elected to use person-first language when referring to disabilities in general (e.g., individuals with disabilities) and identity-first language when discussing the disabled community to which we belong (e.g., D/HH individuals).

*Biopsychosocial models* integrate aspects of individual, social, and diversity models of disability. Engel (1977) originally proposed a biopsychosocial model as a way of addressing an overreliance on a medical model in psychiatry, which ignored the “social, psychological, and behavioral dimensions of illness.” Since that time, the model has been expanded and updated to apply to many areas of health, illness, and disability (Wade and Halligan, 2017). It was used as the basis for the World Health Organization’s International Classification of Functioning, Disability, and Health (ICF), a framework for measuring individual- and population-level health and disability in 2001 (World Health Organization, 2001; Kostanjsek, 2010). At the time, the ICF was seen as progressive, because it included not only the biological underpinnings that give rise to disability (medical model), but also considered that the environment, society, and relationships can exacerbate or ameliorate disability (social model) and indicated that personal factors can also have a major impact on disability (diversity model). While personal factors remain underspecified in the ICF model, these factors could potentially include income, age, educational qualifications, and racial, gender, or LGBTQIA+ identity (WHO, 2011; Shaw et al., 2012; Nakkeeran and Nakkeeran, 2018).

The biopsychosocial model has the potential to be transformative because it acknowledges that there are limitations to our ability to address hearing loss and other disabilities through medicine. By combining medical, societal (i.e., environmental), and diversity considerations we can maximize opportunities for the success of disabled individuals. For example, combining cochlear implants (medical) with accommodations such as closed captioning and training of colleagues/teachers on how to facilitate communication (social) as well as introducing the disabled individual to other disabled peers (diversity) has a higher likelihood of success than any of these strategies in isolation.

## DIVERSITY AND INTERSECTIONALITY

Hearing loss ranges from mild to profound. For example, an individual with mild hearing loss may experience difficulty understanding speech in noisy environments, whereas an individual with profound hearing loss may have little to no functional hearing. Communication modalities found within the hearing loss community can include audio-visual, where speechreading is combined with residual hearing, and visualonly, such as sign language and Cued Speech. The diversity of hearing loss etiologies and communication modes means the hearing loss community is heterogeneous and accessibility strategies for a particular individual (i.e., providing assistive listening devices) may not work for another individual.

Differences in the wording of questions across surveys makes it difficult to estimate the true prevalence of hearing loss (Mitchell, 2006). Nevertheless, rough estimates suggest that approximately 15% of American adults report some trouble with hearing (Blackwell et al., 2012). Global data indicate that ∼6–18% of the world’s population live with some form of hearing loss (Olusanya et al., 2019). For comparison, these numbers are similar to the numbers for diabetes, which affects 13% of American adults (Centers for Disease Control and Prevention, 2020) and 9.3% of adults across the globe (International Diabetes Federation, 2019). Given its high prevalence, it is not surprising that the hearing loss community is inherently diverse. Hearing loss can occur in any individual regardless of race, ethnicity, gender, sexual orientation, socio-economic status, age, or other cultural background. This diversity can contribute to large disparities in terms of rehabilitative outcomes achieved by people with hearing loss. For instance, a child with hearing loss born into an affluent family may have better hearing outcomes due to better parental follow-up from newborn hearing screenings, early intervention such as speech therapy, and earlier access to hearing aids, cochlear implants, or assistive listening devices. A child born into a family of low socioeconomic status might not have access to the same resources (Holte et al., 2012; Ching et al., 2013).

There are strong parallels in the history and consequences of discrimination between individuals with hearing loss and other marginalized communities. Strategies developed to recruit and support members of the D/HH community in STEMM fields can serve as a template for efforts to recruit and support members of other historically disadvantaged or otherwise marginalized communities. The approach presented below is targeted towards D/HH individuals but can easily be adapted for the specific needs of other marginalized groups.

## PROMOTING DIVERSITY

The Association for Research in Otolaryngology (ARO) is a scientific organization focused on hearing and balance research that was founded in 1973. In 1992, D/HH attendees at the ARO Midwinter Meeting formed an informal networking group dedicated to increasing the representation of D/HH individuals in hearing research. This group has grown to more than 110 members globally and has encouraged ARO to develop five pillars that support diversity and inclusion among their members (see Figure 1). These pillars are: 1) fostering peer-mentor groups, 2) proactively providing equal access, 3) easing financial burdens, 4) recruiting for leadership positions, and 5) establishing a culture of inclusion and equity. ARO is a model organization that has successfully implemented practices targeting all five pillars. Below, we provide examples for each that can be used as a framework for other STEMM professional and scientific organizations.

### Fostering Peer-Mentor Groups

Informal interactions between marginalized peers have been shown to promote academic and social growth in higher education settings (Gurin et al., 2002; Tienda, 2013). Diversity networks have the potential to advance D/HH individuals in STEMM who are vastly underrepresented (0.13–0.19%) compared to the general population (11–15.3%; National Science Foundation, National Center for Science and Engineering Statistics, 2021). Over the last ∼30 years, D/HH members and attendees of conferences relating to audiology, otolaryngology, and hearing science have established a distributed academic peer-mentorship network (Adler et al., 2017) to address this disparity. This peer-mentorship network is known as “Hearing Impaired members of ARO” (HI-ARO) due to its founding at an ARO meeting, though the group has expanded to include researchers and clinicians who are involved in hearing science but do not attend ARO meetings. HI-ARO has successfully recruited and retained D/HH scientists and clinicians, growing from three members in 1992 to more than 110 members in 2021. Unlike traditional mentoring, in which senior colleagues mentor junior colleagues in similar positions, peer mentorship refers to formal or informal mentoring among individuals who may be professional equals and/or have different types of jobs (Holbeche, 1996). Peer mentorship can be a valuable tool for underrepresented communities (Cree-Green et al., 2020; Williams et al., 2020) and individuals with disabilities (Hibbard et al., 2002; Thompson et al., 2020; Veith et al., 2006, for a review see: Hayes and Balcazar, 2008). Peer mentorship in our group is informal, occurring at group gatherings at professional society meetings (HI-ARO members comprised ∼1% of total ARO meeting attendees in 2020, or 18 out of 1,798 attendees) and throughout the year via small personal meetings, email, Facebook (@deafearscience), Twitter (@earscience) and our website (www.deafearscientists.org). Most of the mentorship and interactions occur via email, which is ideal for a global group of individuals who often find written communication to be preferable to speaking and listening in a large group.

**FIGURE 2 |:**
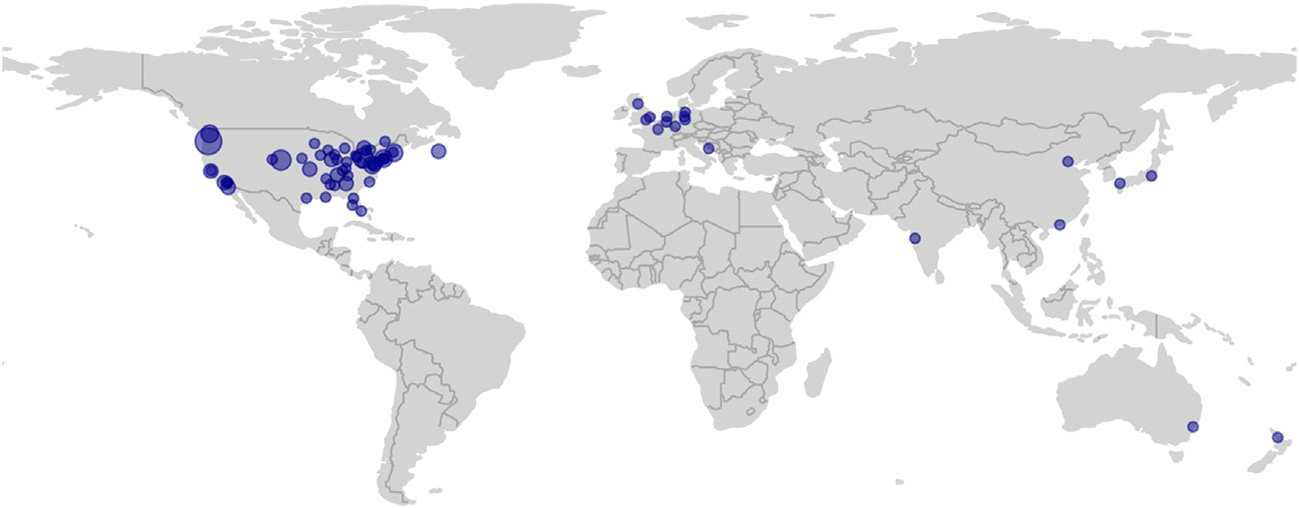
HI-ARO is a diverse group with deaf and hard-of-hearing (D/HH) members from all around the globe. Each dot represents a city that has at least one member of HI-ARO. The dot size is scaled according to the number of members at that location.

HI-ARO includes researchers at all career stages and STEMM disciplines, from trainees to senior scientists, medical students to clinicians, as well as audiologists and other leaders in healthcare and industry. HI-ARO members are highly diverse, spanning race, gender, nationality, socioeconomic status, religion, and disability (see Figure 2 for a map of current members’ locations worldwide). Members have a wide range of unilateral and/or bilateral hearing loss ranging from mild to profound with various etiologies. They use different communication modalities including oral, Cued Speech, and sign language. They may or may not use hearing devices and other augmentative technology. The rich diversity of HI-ARO has been instrumental in cultivating invaluable networking opportunities for D/HH trainees that provide support and guidance from peers and senior members, which is critical to personal and professional success. The diversity within the group has required the members to develop strategies that work equally well for all members, regardless of their mode of disability. For example, despite the large range of communication modalities, captioning was selected as the default method of access that is always provided at conferences. If individuals need other accommodations, such as a cued language transliterator, oral transliterator, or sign language interpreter, these can be requested separately.

### Proactively Providing Equal Access

For scientists, engineers, and other researchers, attending national conferences is important for career advancement. It is at conferences that researchers learn about the work of others, present their own preliminary findings to the scientific community, and network for access to opportunities. However, effective communication can be challenging for attendees due to noisy conference halls, reverberant lecture halls, and suboptimal or inconsistent use of microphones (Atcherson and Yoder, 2002). For D/HH individuals in particular, these challenges can limit the benefits of attending conferences. Frequently, the D/HH attendee needs to personally contact, advocate for, or even explain their disability to conference organizers as they seek accommodations. Often the attendee needs to decide, well in advance of the meeting, what their exact schedule will be so accommodations can be arranged. Sometimes they must find strategies to facilitate communication on their own by carefully choosing listening locations, or using smart phone apps. This creates additional work and stress for D/HH individuals. Beginning ∼20 years ago, ARO shifted from a retroactive approach (i.e., provided on request) to a proactive one where all podium sessions are automatically captioned regardless of whether accommodations were requested. This has not only benefited D/HH attendees but also attendees without hearing loss, especially for non-native English attendees and those who are fatigued after several hours of listening to podium presentations.

In recent years, ARO leaders created an accessibility committee made up of D/HH trainees and faculty to further improve accommodations. This has led to expanding captioning to small group meetings and workshops. Strategies for captioning poster sessions with automatic speech recognition apps and incorporating accommodations that are inclusive of other disabilities, such as color blindness, are being pursued. The widespread prevalence of captioning at ARO has provided D/HH attendees the ability to decide at the last minute what presentations they would like to see without having to worry about whether they would be able to follow the discussion. ARO is currently working on addressing acoustic issues in noisy poster halls by reducing the number of posters in a single room and spacing the posters farther out.

It should be noted that some individuals may need additional accommodations (such as a cued language transliterator or sign language interpreter). The registration form ARO uses has a specific field where registrants can describe the accommodations they need. ARO’s management company will then reach out to the individual to set up the necessary arrangements.

Of particular relevance to many professional societies that host meetings within the United States is the Americans with Disabilities Act (ADA, 1990). The ADA prohibits discrimination against people with disabilities and requires that businesses open to the public, including nonprofit organizations, ensure that people with disabilities have equal access to all that they offer. The ADA typically applies to professional society meetings as they are open to the public (i.e., non-members can attend if they pay a registration fee) and are often partially funded by federal grants. Indeed, the National Institutes of Health (NIH) R13 and U13 funding mechanisms require that the proposal describe strategies for “involving the appropriate representation of women, minorities, and persons with disabilities in the planning and implementation of, and participation in, the proposed conference.”

While the ADA requires equal access for these meetings, there is a difference between the professional society complying with the letter of the law, which places tremendous burden on the disabled individual to advocate for themselves and seek services and being proactive about reaching out to disabled attendees and securing accommodations for them. By being proactive about accommodations, professional societies can nurture and support diversity within their communities while avoiding potential conflicts between disabled individuals and conference management.

The optimal approach may vary from organization to organization. For example, ARO is aware that they have approximately 20 D/HH members from our group attending the annual meeting, along with an unknown number of attendees who may not wish to disclose their hearing difficulties. Thus, the proactive approach for ARO is to automatically arrange for captioning of podium talks, ensure assistive listening devices are available and set up the conference facilities with the goal of improving acoustics. During the registration process, ARO captures information about additional access services that may be required. For other organizations who may feel they do not have a critical mass of D/HH attendees, they can still be proactive by including questions in the registration form to identify anyone who might need special support services. Inclusive practices such as captions and designing the conference with acoustics in mind benefit some members with age-related hearing loss, auditory processing difficulties, or other listening challenges (e.g., listening in a non-native language) but who do not self-identify as D/HH. Finally, being proactive can greatly increase the likelihood of increasing diversity in the organization by encouraging future participation of marginalized individuals.

### Easing Financial Burdens

Increasing access to opportunities for diverse trainees with additional avenues for financial support is essential for cultivating personal and professional career success (Stevens et al., 2021). Many scientific and professional STEMM organizations, including ARO, have travel awards for trainees to attend conferences. For over a decade, the ARO Diversity Committee has used hearing loss status, and other disabilities, as an additional evaluation parameter for the Diversity Travel Award. This has since enabled D/HH trainees to receive financial support to attend conferences, furthering their scientific and professional growth and promoting equity for ARO trainees with disabilities.

### Recruiting for Leadership Positions

The organizational committees of scientific and professional organizations provide an opportunity for trainees, faculty, and other members to take leadership roles within the organization. At ARO, D/HH trainees and faculty have been recruited to multiple committees, including Council, student-postdoc leadership committees, and those that target diversity and accessibility initiatives. For scientific organizations to practice true inclusion and equity, diverse members need to be in organizational and leadership positions. Diverse perspectives at the senior level can yield innovative approaches to drive the organization forward and encourage constructive changes that advance equitable principles. These experiences allow for D/HH individuals to gain the confidence to seek similar leadership roles at their home institutions.

### Establishing a Culture of Inclusion and Equity

The five pillars described above have focused on how scientific organizations can support their diverse members. However, support should also come from other members. Here, organizations have an opportunity to provide tools for all members to learn from diverse perspectives so that they can reflect, grow, and start to advance and advocate for inclusion and equity in their professional and personal lives. ARO has hosted several events aimed at increasing awareness of diverse perspectives, addressing personal biases, and providing tools that help mitigate negative situations for marginalized attendees. These include events where notable individuals with hearing loss share their experiences, roundtable discussions for Women and Allies, networking events, and hiring outside facilitators to provide bystander training for all ARO members. From the point of view of scientific organizations, it can be difficult to assess whether progress has been made in establishing a culture of inclusion and equity. One way organizations can track progress is through anonymous surveys, where members can report whether they feel that the organization has or has not been successful in fostering inclusivity, identify areas of improvement, or voice concern if exclusionary conduct has occurred. This gives the organization an opportunity to improve in areas that are lacking, intervene if there is a problematic situation/person, and track progress of efforts over time. In addition, organizations can track the number of diverse individuals on society committees and leadership roles to ensure that representation is maintained and/or exceeded over time. It should be noted that in other fields, such as deaf education, certain conferences place great emphasis on accessibility. For example, D/HH individuals are included in the meeting planning and keynote presenters are chosen who are themselves D/HH. ARO is working to emulate lessons learned at these conferences by setting up an accessibility committee consisting of D/HH and other disabled individuals. In the past year, greater emphasis has been placed on inviting individuals with hearing loss, such as a deaf comedienne, to present at AROorganized events.

### Broader Impact

Over the last several years, an increasing number of D/HH clinicians and scientists have been presenting at auditory research conferences via posters and podium talks highlighting the need for expanded accessibility. Even within the field of hearing science and related clinical fields, some organizations still resist the need to accommodate a growing number of attendees with hearing loss. Nevertheless, the last decade has seen captioning at the biannual Conference for Implantable Auditory Prostheses (CIAP), the yearly American Cochlear Implant Alliance (ACIA) conference, the American Auditory Society annual conference, and the CI CRASH Midwest MiniConference on Cochlear Implants (hosted by University of Wisconsin-Madison). After three D/HH presenters had trouble understanding and answering questions at a CIAP meeting in the mid-2000s, one attendee declared that CIAP and related conferences should provide captioning. Since then, CIAP has received NIH funding for captioning which in 2019 benefited a record number of 13 D/HH attendees. By proactively seeking funding for captioning and providing access to all attendees, including those who are D/HH, these organizations stand out as truly being inclusive.

Our experience illustrates the proclamation from the Disability Rights Movement, “Nothing about us without us.” D/HH scientists and clinicians should strive to be essential stakeholders in organizations that focus on hearing research and audiology so that the perspectives and experiences of D/HH individuals cannot be ignored. Moreover, equitable access in educational and workplace settings, and at conferences, will help establish productive research trajectories and increased diversity in these settings.

For nearly 30 years, HI-ARO has worked with our professional organizations, especially ARO, to implement successful and practical strategies to increase inclusion of its D/HH members. With the guidance of our peer-network group, ARO leadership has continuously improved accommodations and provided essential support for academic, research, and career development success for D/HH members. ARO’s support for equity and diversity has served and may continue to serve as a model for other conferences and professional organizations.

## FUTURE DIRECTIONS

This article has described a highly successful model for promoting representation of D/HH individuals in hearing research. We have found that D/HH individuals have used their early career in hearing research, combined with mentorship from our group, as a stepping stone to other fields of study and medical specialties. This same support needs to be extended to inclusion of other disabilities, such as visual impairment, in their chosen field, and to the inclusion of D/HH individuals in STEMM fields beyond hearing research. Funding opportunities from the National Science Foundation (NSF 21-049, NSF 21-110) and the National Institutes of Health (R13, U13, R25) gives professional societies financial support to implement the five pillars, such as with conference costs (e.g., captioning) and mentoring activities.

The fact that the strong D/HH network arose in the auditory field is not surprising. After all, auditory researchers and clinicians understand communication challenges and many of us have been fortunate to have been mentored by normal hearing individuals who advocated for access within ARO. However, many other scientific societies have unfortunately not yet followed the lead of ARO. To fully realize equal participation of D/HH individuals in STEMM will require administrative champions in the leadership of other societies given the cost of assistive technology. The hearing research field is relatively small, and STEMM will benefit when D/HH individuals have full access to career opportunities in the field of their choice-not limited by the accessibility of the field or others’ perceptions of what would be a “natural” choice based on their disability. Thus, the scientific community needs to come to this realization and do more for D/HH individuals across disciplines.

Professional organizations, including those that are not STEMMfocused, can include individuals with disabilities by inviting them to provide input on policies such as disability accommodations at scientific meetings. The COVID-19 pandemic provided an opportunity to reshape accommodations for those with disability when in-person conferences were shifted to virtual. For a virtual poster, a presentation is often recorded, without background noise, and can easily be captioned. Digital platforms can facilitate asynchronous text-based research interactions. This mode can be easier for many D/HH individuals and other individuals with disabilities than in-person sessions and conversations in noisy conference halls. Hybrid conferences adopting universal design approaches combined with remote manual correction and formatting of automatic captioning might proactively reduce isolation while still affording these benefits.

Another opportunity for professional organizations to foster diversity is to create mentoring programs for diverse middle school and high school students, including those with disabilities. Many students from historically excluded groups have the potential to succeed in STEMM fields but do not always receive the guidance and support needed to pursue those paths. Having a mentor who has faced similar challenges can bring confidence in learning and self-advocating and provide encouragement and inspiration. Existing models such as AG Bell’s Leadership Opportunities for Teens (LOFT), Oregon Health Science University’s On Track OHSU! and others (Pluth et al., 2015) provide a framework for adopting similar programs for STEMM outreach.

Our peer-mentorship network of D/HH engineers, scientists, and clinicians is foundational to our success in STEMM. Trainees have successfully transitioned to being leaders in academia, healthcare, and industry, often receiving significant research funding. Through this network we discuss the challenges we face and strategies to overcome difficulties. We also form a cohesive group to advocate for changes in our fields and at professional conferences to address the underrepresentation of individuals with hearing loss in STEMM. We believe that the adoption of this peer-mentorship network model by other underrepresented minority groups, and collaboration among and between peer-mentorship networks and professional societies, will drive changes that will promote diversity and equity in STEMM and other fields.
